# Editorial: Immune Cells in the Mucosa

**DOI:** 10.3389/fimmu.2016.00657

**Published:** 2016-12-26

**Authors:** Pushpa Pandiyan, Ed C. Lavelle

**Affiliations:** ^1^Department of Biological Sciences, School of Dental Medicine, Case Western Reserve University, Cleveland, OH, USA; ^2^Adjuvant Research Group, School of Biochemistry and Immunology, Trinity College Dublin, Trinity Biomedical Sciences Institute, Dublin, Ireland

**Keywords:** mucosal immunity, infection, HIV, periodontitis, IBD

**Editorial on the Research Topic**

**Immune Cells in the Mucosa**

## Intestinal Immune Cells and Autophagy

The importance of autophagy as a key regulator of cellular homeostasis and responsiveness to stress and infection is being increasingly appreciated as reflected in the recent award of the Nobel Prize in Physiology or Medicine to the Japanese researcher Yoshinori Ohsumi (https://www.nobelprize.org/nobel_prizes/medicine/laureates/2016/ohsumi-facts.html). In the review by Kabat et al. titled “*The Mucosal Immune System and Its Regulation by Autophagy*,” the emerging importance of autophagy as a regulator of mucosal immunity is addressed. The pathway plays a key role in a number of aspects of intestinal immunity including epithelial barrier function and the inflammatory properties of intestinal mononuclear phagocytes. Autophagy also plays a role in the maintenance of peripheral CD4^+^ and CD8^+^ T cells and memory CD8^+^ T cells and influences the balance of intestinal T helper cell subsets through mechanisms related to metabolic regulation. The authors speculate that autophagy plays a key role in endowing T cells with the metabolic flexibility to adapt to challenges relating to growth factor and nutrient availability. As a result, the effects of autophagy modulation on immune responses depend on the specific cell type and tissue context. Finally, the authors address the exciting potential for therapeutic modulation of autophagy as a strategy for treating inflammatory bowel disease (IBD) and other chronic conditions.

## Gut Mucosal Immunity and Microbiome in IBD

Homeostatic mechanisms ensuring appropriate immune responses against invading pathogens while preventing unwanted immune responses against innocuous antigens such as food and gut resident microbiota are characteristic features of gut mucosal immunity. Dysregulation of these homeostatic mechanisms and aberrant immune responses toward intestinal bacteria characterize IBD. In a review titled “*Mucosal Interactions between Genetics, Diet, and Microbiome in Inflammatory Bowel Disease*,” Basson et al. emphasize pantropic mechanisms in the context of inter-relationships among mucosal immune cells, disease-associated genes, dietary factors, and gut microbiota composition, rather than univariate connection between each of these axes. They review some of the studies investigating (1) host genetics in altering gut microbiome, (2) the interdependence between dietary factors and gut microbiome, (3) microbiota-derived fatty acids, (4) how bile acids affect gut microbiota, and (5) immune modulatory and immune stimulatory effects of intestinal micronutrients, toxins and chemicals.

## Antigen-Presenting Cells in Airway Mucosa

In “*Unique Trans-compartmental Bridge: Antigen Presenting Cells Sampling across Endothelial and Mucosal Barriers*,” Allen et al. review recent data on the ability of mononuclear cells at the intestinal and other mucosal surfaces to sample pathogens across an intact epithelial barrier. This remarkable process has been principally revealed by advances in intravital microscopy. Evidence suggests that dendritic cells in the lamina propria and Peyer’s patches can extend dendrites that contact and facilitate the uptake of bacteria across the epithelial lining. This allows sampling of the lumen and can result in the induction of antigen-specific immunity. The authors draw an interesting parallel between this behavior and comparable sampling processes by antigen-presenting cells in secondary lymphoid tissues. These findings include the demonstration that lymph node resident dendritic cells can extend dendrites into lymphatic fluid to sample antigens and this can initiate early T cell activation. Evidence for transcompartmental sampling of antigens by dendritic cells in the airway mucosa and at the blood brain barrier is also presented, but here additional studies are required to further substantiate the functional significance of the findings.

## CD4^+^ T Cell Immune Responses to Pneumocystis Pneumonia in Lung Mucosa

In their article “*CD4^+^T-Cell Independent Secondary Immune Responses to Pneumocystis Pneumonia*,” de la Rua et al. present new data on protective immunity against *Pneumocystis* pneumonia that causes significant morbidity and mortality in immunocompromised patients, particularly in the context of human immunodeficiency virus (HIV)/AIDS. The authors focus on the roles of specific immune cell populations and antibodies in the memory recall response to infection. They demonstrate that while CD4^+^ T cells are dispensable, mice depleted of CD8^+^ T-cells or alveolar macrophages had significantly higher fungal burden in the lungs following challenge. In addition to these responses, IgG from infected animals significantly increased the killing of *Pneumocystis murina* by macrophages. The results point to essential roles for alveolar macrophages, CD8^+^ cells and antigen specific IgG in mounting protective recall responses to infection with this opportunistic fungal pathogen.

## Oral Mucosal B Cells in Periodontitis

Although periodontopathic bacterial species such as *Porphyromonas gingivalis* and *Aggregatibacter actinomycetemcomitans* are the initial perpetrating agents, subsequent progression and severity of periodontitis are determined by immune responses in gingival mucosa. Mucosal adaptive immunity involving antigen-specific activation of T and B cells is strongly associated with periodontal tissue destruction in experimental animal models and humans. Immunosenescence associated with aging also plays an important role in the disease. Although extensive literature on periodontitis has documented end results of adaptive immune functions, little or no information is available regarding adaptive immune cellular changes occurring during aging and progression of periodontitis in mucosal tissues. Here, Ebersole et al. in their article titled “*Transcriptome Analysis of B Cell Immune Functions in Periodontitis: Mucosal Tissue Responses to the Oral Microbiome in Aging*” took a transcriptome profiling approach, comparing healthy, aged, and periodontitis gingival samples from humans and non-human primate models. They report substantial transcriptome alterations affecting a large number of genes critical for antigen-dependent activation, proliferation, T-cell interaction, and maturation of B cells in periodontitis in adults and aged animals. They conclude that with healthy aging, adaptive B cell responses modulate tissue destructive gene expression and maintain oral mucosal immune homeostasis. However, such homeostatic B cell activities are lost, resulting in immundysfunction and enhanced inflammatory responses during periodontitis.

## Mucosal T Cells in HIV-Associated Immune Activation

Residual mucosal inflammation along with chronic systemic immune activation is an important feature in individuals infected with HIV. These conditions have been linked to a wide range of comorbidities, including malignancy, opportunistic infections, and cardiovascular complications in patients on anti-retroviral treatment. T_reg_ and Th17 cells are closely related CD4^+^ T cells, sharing a reciprocal relationship and playing central roles in controlling human inflammatory diseases. Reduction of systemic and gut/rectal mucosal Th17 cells and T_regs_ (despite increased T_reg_/Th17 ratio) has been strongly associated with gut microbial translocation and systemic inflammation during HIV infection. However, the majority of research on immune activation has been derived from analysis of circulating immune cells. How immune cell alterations in mucosal tissues contribute to HIV immune dysregulation and the associated risk of non-infectious chronic complications is less studied. We currently have little or no understanding on HIV-associated immune dysfunction, and its relationship to T_reg_ and Th17 cells in other mucosa. In an article entitled “*Mucosal Regulatory T Cells and T Helper 17 Cells in HIV-Associated Immune Activation*,” Pandiyan et al. present a hypothesis that the pro-inflammatory milieu in HIV^+^ patients with immune activation contributes to enhanced loss of Th17 cells and increased frequency of dysfunctional T_regs_ in the mucosa, which in turn may exacerbate immune dysfunction in HIV-infected patients. They present some evidence to support this hypothesis. Using transcriptome profiling of CD4^+^ T cells in peripheral blood mononuclear cells, they demonstrate increased pro-inflammatory cytokine signaling in HIV^+^ patients with higher immune activation. They discuss how the pro-inflammatory milieu might impact T_reg_ and Th17 cells in the gut and oral mucosa. Future studies along this line will shed light on mucosal immune dysfunction and HIV reservoirs in tissues and lead to novel ways to restore immune functions in HIV^+^ patients.

## Author Contributions

PP and EL contributed equally to this work.

## Conflict of Interest Statement

The authors declare that the research was conducted in the absence of any commercial or financial relationships that could be construed as a potential conflict of interest.

